# Abuse and other correlates of common mental disorders in youth: a cross-sectional study in Goa, India

**DOI:** 10.1007/s00127-012-0614-6

**Published:** 2012-10-31

**Authors:** Andrea C. Fernandes, Richard D. Hayes, Vikram Patel

**Affiliations:** 1 Institute of Psychiatry, King’s College London, London, SE5 8AF UK; 2 Sangath Centre, 841/1 Alto Porvorim, Goa, 403521 India; 3 London School of Hygiene and Tropical Medicine, Keppel Street, London, WC1E 7HTK UK

**Keywords:** Youth, 16–24 years, Common mental disorders, India, Community survey

## Abstract

**Purpose:**

There is a paucity of known correlates of common mental disorders (CMDs) among the youth age group in India. This analysis aims to determine risk factors associated with a probable diagnosis of CMD in a youth sample in India.

**Methods:**

This is a secondary analysis of data collected via a door-to-door (community) survey of 3,662 youth (aged 16–24 years) in selected urban and rural areas in Goa. The urban and rural areas were selected based on their engagement with a Goan-based mental health charity organisation, Sangath. Point prevalence of CMD was estimated using the general health questionnaire-12 (GHQ-12). Multivariate logistic regression analyses determined factors associated with CMD and associations were stratified by gender.

**Results:**

In total, 3,649 (1,796 urban; 1,853 rural) youth were assessed for probable diagnosis of CMD. There was an almost equal ratio of males (49 %) to females (51 %) in the sample. During the time of the survey, 91 % of the sample was residing with parents, with 83 % being between the ages of 22 and 24 years living with parents. A small proportion of the sample never attended school (1.1 %) with the rest either educated, employed or unemployed. The point prevalence of probable CMD in the sample was 7.87 %; 95 % CI 7.01–8.80 %. Those living in urban areas had a higher prevalence of CMD (9.12 %; 95 % CI 7.90–10.52 %) compared to those living in rural areas (6.60 %; 95 % CI 5.50–7.82 %). After adjusting for a range of potential confounders, independent risk factors for CMD were being older, i.e., between 22- and 24-years old, (OR 1.60; 95 % CI 1.10–2.24; *p* = 0.015), residing in urban areas (OR 1.51; 95 % CI 1.12–2.04; *p* = 0.007), physical abuse (beaten in the last 3 months) by parents, teachers or others (OR 3.10; 95 % CI 2.11–4.51; *p* < 0.001), sexual harassment (OR 2.01; 95 % CI 1.30–3.20; *p* = 0.003) and sexual abuse (OR 2.54; 95 % CI 1.94–3.33; *p* < 0.001). Being able to talk about personal problems (OR 0.52; 95 % CI 0.34–0.80; *p* = 0.003) was a protective factor. After stratifying by gender, sexual harassment, physical and sexual abuse were associated with a likely CMD diagnosis in females and males.

**Conclusions:**

Sexual and recent physical abuses were independent risk factors for CMD in both genders. In addition, being older and being able to discuss problems were associated with CMD diagnosis in females but not in males.

## Introduction

In the 1970s, research showed increasing trends in rates of depression among young people [1]. Forty years on, common mental disorders (CMDs), defined usually by depression (including unipolar major depression), anxiety and somatoform disorders [2–5], continue to have an impact on the lives of young people. Based on the 2004 WHO global burden of disease report, Gore et al. [6] reported that in young people (aged 10–24), the leading cause of years lost due to disability was attributed to unipolar depressive disorders (ranking first among all the contributors to disability (mental or physical) with accidents, violence and serious mental disorders following suite). The first nationally representative survey of adolescents suffering from mental disorders (published only in 2010) in the United States showed that anxiety disorders were the most frequently occurring and that one in five adolescents experience symptoms of mental disorders [7].

Although research focusing on CMD in young people is growing [8, 9] it is still very limited, in particular among the youth (i.e., 15–24) age group [10], in many lower and middle income countries (LAMICs) where communicable forms of diseases and reproductive health have traditionally been given priority [6, 9]. It is projected that 20 years from now (in 2032), 90 % of the global population of young people aged 10–24 years will be living in LAMICs [6]. There is an urgent need to understand CMD prevalence and correlates specific to young people in LAMICs.

Youth is defined officially by the UN to be between the age range of 15 and 24 [11] and is also known as the transition phase in which individuals go from being an adolescent to becoming a young adult. Life events, such as applying for university, new found freedom and exploring relationships, taking place during this age may well differ from child, adolescent and adult populations [6, 12–15]. Research indicates that many mental disorders that manifest during adulthood have first onset during youth and hence understanding and detecting mental illness at this stage could mean preventing further deterioration of mental health [6, 9]. Risk factors of CMD such as family history of depression, bereavement, break up of romantic relationships, chronic medical condition; physical and sexual abuse, trauma and severe stress, anxiety, learning difficulties and substance abuse have been established as associated with CMD among youth [16, 17]. In a review, Gilbert et al. [18] showed that maltreatment (includes sexual and physical abuse) during childhood has a 1.3–2.4 times increased likelihood of being associated with depression in adulthood. If not associated with internalising forms of mental health, abuse can affect education, social behaviours, suicidal ideation and self-esteem [18].

India is home to the second largest population in the world, with youth projected to form one of the highest proportions of the population in 2030 [19] and research on CMD in India among this age group specifically is limited [20–23]. In India, a lot of evidence on correlates of CMD is based on adolescent and adult population and not much dedicated to the transition youth age group, except for two studies reporting a 6.6 % prevalence of CMD in all female samples (aged 18–24) in Goa [24] and the other study reporting a prevalence of depression and anxiety of 6.4 and 5.4 %, respectively, in an all male cohort (*n* = 500, aged 17–24 years) in Bihar [25]. A review on prevalence of psychiatric disorders (including CMDs) among child and adolescents (ages ≤19 years) in India identified 55 studies reporting widely varying prevalence estimates ranging from 0.62 to 41.90 % [20].

Given the growth of the youth population in India, we aimed to strengthen the evidence based on CMD and risk factors in this age group by investigating correlates of CMD from the largest population-based youth (16–24) community survey (including rural and urban areas).

## Methods

### Study design, settings and recruitment

This investigation was conducted in the Indian state of Goa. Ten urban wards (a ward is the division of a city or a village into smaller geographical areas for administrative purposes) and four rural villages were chosen as the setting for an exploratory Randomised controlled trial (RCT) to promote the health of young people [26]. The survey conducted prior to the start of the trial formed the baseline data, which we used to conduct this cross-sectional analysis of CMD and its correlates.

Recruitment took place between March and July 2006 by trained researchers through face-to-face interviews. Before the recruitment process, study awareness programs were conducted in both rural and urban areas. These included meetings in the community organized through panchayats (self-government units at village/small town level) and other key groups such as religious groups and sports clubs. This also included conducting games and teaching hobbies in the communities and requested the cooperation of young people for the study. A door-to-door survey was conducted in ten urban wards (total population 34,565) and four rural villages (total population 14,794). Around 8 % of the urban population sample was in the youth age group while in the rural villages, around 14.9 % made up the youth population sample. All youth were eligible for the study except those who had significant visual impairment, hearing disability, intellectual disability, or who could not communicate in one of the three study languages were excluded (*n* = 5). Youth in urban and rural communities that were enumerated and those who were available (67 % of youth in urban and 81 % in rural) received a verbal introduction to the study by the researcher was provided with an information sheet, and then approached for consent to participate in the study. The main reason for non-participation was that youth were working or studying elsewhere (so most of the non-participants were not resident in the study setting and it was not possible to gather information on the non-participants). Among those approached for consent, refusal rates were 1.2 % in urban and 5.2 % in rural. After the survey, 3,649 (rural *n* = 1,796; urban *n* = 1,853) had data on CMD collected; this was the final sample size used for this data analysis. Data collected were entered into SPSS version 17.0, and were cross checked for any error in data input or duplicate cases.

### Measures

A structured interview was developed specifically for the survey. The interview was based on previous research studies, including a study on the health needs of adolescents in schools, a population-based study of mental health in young adolescents and a population-based cohort study of women’s reproductive and mental health [24, 27]. The survey was separated into sections: Socio-demographic profile, education, career choices, interpersonal relationships (including being beaten), emotional health, general health questionnaire with 12 items (GHQ-12), self harm, harming others, substance abuse, reproductive health, sexual relationships, sexual violence and general help seeking.

#### Main outcome

The outcome of interest was having a probable diagnosis of CMD which was measured using the GHQ-12. Individuals could score a maximum of 12 points. Those scoring 5 and above were considered to have a higher likelihood of CMD. For validity, the instrument, initially developed in English, was translated into the other two local languages via a standard translation and back-translation process. The instrument was then piloted, for clarity and face validity, among 87 young people from a comparable but different community to assess its acceptability and feasibility [26]. There is only one formal validation study of the GHQ from Goa which was carried out with adult primary care attenders, and which identified the cut-off point of 5 as having the lowest misclassification rate [28]. Patel and colleagues [28] reported that the GHQ-12 cut-off score of 5/6 showed optimal validity (73 % sensitivity; 90 % specificity; 61.2 % positive predictive value) relative to other cut off scores in a Goan sample. The study sample included young adults and we have therefore adopted this same cut point in the current study. A cut-off score of 5/6 was used in two other reports [29, 30] based on the same study [26] on Goan youth aged between 16 and 24, while another report (of the same study) used a cut-off score of 3/4 [26].

#### Explanatory variables

The data collected from the survey administered to the sample allowed us to look at several factors for their potential association with CMD. These factors were chosen based on previous literature. The association of CMD with suicide [30] and violence towards others [29] using the same dataset were reported elsewhere and were not explored here. The following factors were explored in this analysis:

#### Demographic and socio-economic factors

Age, gender, education, area of residence (urban or rural) and socio-economic status. The socio-economic status was assessed based on an asset index score which was generated from an item in the survey questionnaire—“Does your household own any of the following: fan, radio, television, telephone, fridge, sewing machine, bicycle, motorcycle, car or tractor”. Respondents indicated if they did (scored as 1) or did not (scored as 0) own each asset. These scores were then summed together to give combined score (maximum 10). These scores were then grouped into tertiles: 0–3; 4–6; 7–10. A high score indicated a higher socio-economic status relative to low scores. Assessing socioeconomic status based on ownership of household assets is consistent with prior work on both international and Indian survey data.

#### Social relationship factors

Marital status; level of autonomy (being able to make decisions—yes, no, sometimes); being able to talk about personal problems with peers, parents or teachers and being able to talk about sex-related issues with peers, parents or teachers (a lot, a little or not at all).

#### Sexual harassment, physical and sexual abuse

Ever been talked to about sex in a way that was uncomfortable (yes, no), recently being beaten (being pushed, grabbed, slapped, hit, kicked, punched or did similar action by teachers, family members or others in the last 3 months—yes, no) and ever being a victim of sexual abuse (touched or fondled your private parts against wishes, showed their sex organs to you against wishes, or forced you to have sexual intercourse—yes, no).

### Statistical analyses

Logistic regression models were constructed, where having a probable diagnosis of CMD was the outcome. Associations between CMD and each of the variables described above were assessed in crude and gender-age adjusted models. Fully adjusted analyses were carried out to identify factors that were independently associated variables with CMD. Odds ratios (ORs) and 95 % confidence intervals (CI) were presented. A final analysis was carried out with the independent variables adjusted for demographic variables and stratified by gender to identify differences among gender. *P* for trend was reported for age and asset index. Data were analysed using STATA/IC version 11.2.

## Results

### Characteristics of the sample

The sample was made of 3,649 individuals between the ages of 16–24 years old with the average age being 19.5 (SD ± 2.50) years. Rural and urban residents were equally represented in the sample: urban (50.80 %) and rural (49.22 %); as was gender: female (51.30 %) and male (48.73 %) (Table 1). In terms of education, 43.60 % of the sample was currently studying, 1.15 % never had an education and 63.61 % of those currently studying had chosen a career. The proportions of males and females currently in education were similar: males (48.73 %) and females (51.30 %). A small proportion (6.60 %) of the sample were married. During the time of the survey, 91.01 % were residing with parents, with 83.73 % of individuals between the ages of 22–24 living with parents. In terms of risky behaviour, 87.80 % did not smoke ever, 99.60 % never took drugs and 88.05 % did not ever have sexual relationships.

**Table 1:** Crude, age-gender adjusted and fully adjusted logistic regression analyses of potential risk factors for a likely diagnosis of CMD, *n* = 3649; probable CMD, *n* = 287

Risk factors	*N*	CMD prevalence (%)	Crude odds ratio (95 % CI)	Gender and age adjusted odds ratio (95 % CI)	Fully adjusted odds ratio^a^ (95 % CI) *n* = 3,629	*p* value^a^
Total sample	3,649	287 (7.87) 95 % CI 7.01–8.80				
*Demographics*						
Age						
16–18	1,488	106 (7.12)	Referent	Referent	Referent	0.018^b^
19–21	1,250	93 (7.44)	1.10 (1.00–1.40)	1.05 (1.00–1.40)	1.12 (0.82–1.53)	0.464
22–24	911	88 (9.66)	1.40 (1.04–2.00)	1.40 (1.04–2.00)	1.60 (1.10–2.24)	0.015
Gender						
Male	1,778	132 (7.42)	Referent	Referent	Referent	–
Female	1871	155 (8.28)	1.13 (1.00–1.43)	1.13 (1.00–1.44)	1.21 (0.93–1.60)	0.150
Area of residence						
Rural	1,796	118 (6.57)	Referent	Referent	Referent	–
Urban	1,853	169 (9.12)	1.43 (1.12–1.82)	1.50 (1.20–1.90)	1.51 (1.12–2.04)	0.007
*Socioeconomic status*						
Number of household assets owned (*Asset index)*						
0–3	1,130	80 (7.08)	Referent	Referent	Referent	0.058^b^
4–6	1,227	118 (9.62)	1.40 (1.04–2.00)	1.40 (1.03–2.00)	1.11 (0.80–1.54)	0.532
7–10	1,292	89 (6.89)	1.00 (0.71-1.33)	1.00 (0.72–1.40)	0.70 (0.50–1.04)	0.076
Currently studying						
Yes	1,591	112 (7.04)	Referent	Referent	Referent	–
No (including never went to School)	2,058	175 (8.50)	1.23 (1.00–1.60)	1.11 (0.84–1.50)	1.14 (0.84–1.60)	0.410
*Social relationships*						
Marital status						
No	3,409	266 (7.80)	Referent	Referent	Referent	–
Yes	2,400	210 (8.75)	1.13 (0.71–1.80)	0.94 (0.60–1.53)	0.93 (0.60–1.60)	0.788
Autonomy (making own decisions)						
Yes all the time	1,099	800 (7.28)	Referent	Referent	Referent	–
Yes sometimes	1,475	124 (8.41)	1.20 (1.00–1.60)	1.20 (1.00–1.60)	1.21 (1.00–1.70)	0.224
No	1,075	830 (7.72)	1.10 (0.80–1.50)	1.10 (0.80–1.50)	1.20 (0.84–1.70)	0.334
Being able to talk about issues related to sex to peers, parents or teachers						
No	1,012	70 (6.92)	Referent	Referent	Referent	–
Yes	2,624	217 (8.27)	1.21 (0.92-1.61)	1.23 (0.93-1.62)	1.33 (1.00-1.80)	0.067
Being able to talk about personal problems to peers, parents or teachers						
No	261	30 (11.49)	Referent	Referent	Referent	–
Yes	3,374	257 (7.62)	0.64 (0.43–0.95)	0.64 (0.43–0.95)	0.52 (0.34–0.80)	0.003
Sexual harassment, physical and sexual abuse						
Sexual harassment (ever been talked to about sex uncomfortably)						
No	3,233	213 (6.59)	Referent	Referent	Referent	–
Yes	414	74 (17.87)	3.10 (2.32–4.11)	3.1 (2.4–4.2)	2.25 (1.63–3.1)	<0.001
Having been beaten in the last 3 months						
No	3,430	244 (7.11)	Referent	Referent	Referent	–
Yes	215	430 (20)	3.30 (2.30–4.70)	3.60 (2.5–5.20)	3.01 (2.05–4.42)	<0.001
Having ever been sexually abused						
No	3,270	222 (6.79)	Referent	Referent	Referent	–
Yes	377	65 (17.24)	2.86 (2.20–3.86)	2.88 (2.14–3.90)	2.06 (1.48–2.87)	<0.001

^a^Fully adjusted model (includes socio-demographics factors, social relationships and physical abuse factors)

^b^ *p* for trend

### Prevalence and variable associations with CMD

The point prevalence of CMD in the sample was 7.87 %; 95 % CI 7.01–8.80. Those living in urban areas had a significantly higher prevalence (9.12 %; 95 % CI 7.90–10.52) compared to those living in rural areas (6.60 %; 95 % CI 5.50–7.82) (Table 1).

Factors that remained associated with CMD after multivariate analysis (Table 1) were age (being older), area of residence, being able to talk to peers, parents or teachers, ever being sexually harassed, physically abused (in the last 3 months) and being sexually abused. All variables that showed a significant association in the full multivariate model were then included in a final model which was stratified by gender (Table 2). In females, risk factors were higher age (OR 1.70; 95 % CI 1.01–2.72; *p* = 0.046); sexual harassment (OR 2.50; 95 % CI 1.60–4.00; *p* value < 0.001); sexual abuse (OR 2.60; 95 % CI 1.80–3.82; *p* value < 0.001) and physical abuse (recently being beaten) (OR 4.10; 95 % CI 2.44–6.90; *p* < 0.001). Being able to talk about personal problems (OR 0.50; 95 % CI 0.30–0.90; *p* value = 0.014) had a protective effect on CMD in females. In males, risk factors were sexual harassment (OR 2.01; 95 % CI 1.30–3.20; *p* = 0.003), sexual abuse (OR 2.45; 95 % CI 1.70-3.60; *p* value < 0.001) and physical abuse (OR 2.23; 95 % CI 1.30–4.00; *p* value = 0.007). Area of residence lost significance after gender stratification (Table 2).

**Table 2:** Final multivariate model presenting all factors significantly associated with CMD in full adjusted model stratified by gender

Risk factors^a^	Male (*n* = 1,776)	*p* value	Female (*n* = 1,853)	*p* value
Being able to talk about personal problems to peers, parents or teachers				
No	Referent	–	Referent	–
Yes	0.71 (0.40–1.32)	0.277	0.50 (0.30–0.87)	0.015
Having been beaten in the last 3 months				
No	Referent	–	Referent	–
Yes	2.20 (1.21–3.90)	0.009	4.00 (2.40–6.68)	<0.001
Having ever been sexually abused				
No	Referent	–	Referent	–
Yes	1.85 (1.20–3.00)	0.009	2.30 (1.43–3.65)	0.001
Having ever been sexually harassed				
No	Referent	–	Referent	–
Yes	2.01 (1.30–3.20)	0.003	2.50 (1.60–4.00)	<0.001
Age				
16–18	Referent	–	Referent	–
19–21	0.80 (0.50–1.30)	0.335	1.50 (1.00–2.23)	0.076
22–24	1.33 (0.80–2.20)	0.268	1.70 (1.00–2.72)	0.050
Area of residence				
Rural	Referent	–	Referent	–
Urban	1.50 (0.94–2.30)	0.092	1.40 (0.92–2.72)	0.143

^a^Adjusted for being able to talk about personal problems to peers, parents or teachers; having been beaten in the last 3 months; having ever been sexually abused and socio-demographic variables (age, gender, education, asset index)

## Discussion

This study looked at factors potentially associated with a probable diagnosis of CMD within a sample of youth in Goa, India. Urban residence, being older, being sexually harassed and abused, being physically abused and being able to discuss problems was associated with CMD after controlling for a number of potential confounders. Sexual abuse and physical abuse in recent months were independent risk factors for CMD in both genders. In addition, being older and being able to discuss problems associated with CMD diagnosis in females but not in males. This was the largest community-based youth survey in India to date. The large sample size enabled us to examine a variety of covariates in the same model.

### Limitations

This study has limitations worth noting. The cross-sectional design does not make it possible to determine the direction of causality and hence the possibility of reverse causality cannot be eliminated. Whether symptoms of CMD existed before the exposure of risk factors or the resultant exposure to risk factors was due to the onset of CMD cannot be deduced. There may be a greater risk of misclassification probable cases of CMD, using a GHQ-12 with a cut-off score of 5/6, as the current study was carried out in a community sample, while the cut-off score of 5/6 was validated in a clinical sample. However, given that there is only one formal validation study of the GHQ from Goa [28], which included young adults this was the cut-off score most valid. Recall bias cannot be eliminated given the questionnaire consisted of several sections enquiring past life events. Several questions (for example on substance abuse and having sexual relationships) may have been answered according to social norms in India. Based on previous literature [31], substance abuse could have potentially been associated with CMD but could not be explored in this dataset as only 16 individuals reported ever having taken drugs. The number of youth that participated in the study was relatively lower in the urban community compared to the rural community (67 vs. 81 % respectively). This could indicate limited generalisability of the findings in the urban sample to the whole of the urban youth population in Goa. As mentioned in the methods due to unavailability (because of study or work elsewhere) we cannot further explore difference in non-participants and participants and it is possible that our sample may not be representative of youth with higher education or qualifications. However, it is accepted that Goa is not representative of the entire population of India—Table 3 (as, indeed, no other single Indian state can be considered representative of the rest of the country) and hence, our findings may not be representative of the entire Indian population.

**Table 3:** Comparison of demographics in percentages: Goa-wide and India-wide statistics (ages from 20–24 years) [60] (Statistics from 2005/06)

	Study sample (%)		Goa* (%) [60]	India (%) [60]		Whole of India (%) [60]
	Urban	Rural		Urban	Rural	
Gender						
Male	40.1	59.9	NA	38.5	61.5	NA
Female	55.1	44.9	NA	32.5	67.5	NA
Illiterate (20–24)						
Female only	0	0.3	8.8	16.9	44.40	35.40
Never married (20–24 years)						
Female only	89.2	78	68.6	38.7	17.8	24.70

* includes urban and rural

In this sample the prevalence of CMD was 7.87 % which is less than the prevalence reported in other studies on youth studies outside of India [32–39] but it does fall within prevalence rates among young and adult samples reported in India [20, 24, 30]. National reports and systematic reviews produced in India commonly show a varied prevalence of mental disorders across India in the adult populations and a low prevalence compared to studies globally [20, 22, 40, 41].

Urban area of residence was independently associated with a higher risk of developing a CMD in this sample (Table 1); this association disappeared after gender stratification possibly due to loss of statistical power (Table 2). This is the second time that urbanicity was found to be a risk factor in the same setting but a different age group. Pillai et al. [27] reported an increased association of urban living and CMD in an adolescent age group in Goa with an odds ratio of 2.2 (*p* = 0.04) compared with adolescents living in rural areas. Increased prevalence and significant association of CMD with urban areas are well known in India though reasons remain to be established. Reddy and Chandrashekar [41] reported, from their meta-analytical review (specific to studies in India), that depression, mental retardation, neurotic disorders and behavioural/emotional disorders were significantly high in urban communities while comparatively less CMD like hysteria and epilepsy were high in rural areas. A national report on mental health research in India has dedicated a chapter on urban and rural differences in mental health in India showing that there is awareness [42] in India that urban and rural areas may differ in risk factors for mental disorders. However, Bhola [20] argues that definite conclusions cannot be made about rural–urban differences due to the wide difference in research methods. Globally, there are mixed findings in the limited literature on mental disorders and area of residence among the general population [43–45]. More comparative investigations, with factors in urban and rural areas, are warranted to clarify the association why urbanicity shows consistent associations with increased risk of CMD among young people [46].

The independent effect of sexual and physical abuse on CMD that is well established in the literature was replicated in this sample [24, 47]. In our sample, individuals who were sexually or physically abused and sexually harassed were two to three times as likely to have probable CMD. A review by Ribeiro and colleagues [47] on the effects of violence on mental health observed odds ratio being twice as high of having mental health problems among children and adolescents who have experienced domestic violence compared to those who did not. This review drew out women and young people as the most vulnerable groups to mental health problems after experiencing physical or sexual violence and pointed out the lack of investigation in males and exposure to violence in communities [47]. Our findings, that physical and sexual abuse affected male youth as much as female youth, contributes to the comparatively less literature among young males and abuse in the general population. Ribeiro et al. [47, 48] highlights that being male puts individual at a higher risk of victims of homicidal violence and violence is usually experienced in the community in the form of accidents, witnessing violence, etc. Literature also suggests that when exposed to violence, women are more prone to mental health problems compared to men. In our study, we found an almost equal risk of having CMD if ever exposed to harassment, physical or sexual abuse in both genders (Table 2). Martin et al. showed, in a prospective study using a sample (*n* = 2485) of 14-year-old adolescents who experienced sexual abuse, that 55.5 % of boys who were abused had some form of suicidal ideation compared to 27 % of females who were abused. Furthermore the study showed that, in males, suicide attempts were associated with abuse even after adjusting for depression and hopelessness (OR 18.7; 95 % CI 5–70.1; *p* value < 0.001), while in females, suicidality was mediated by depression (non-significant OR reported, data not presented) [49]. There are currently no studies in India exploring the potential effects on mental health among male victims of abuse [50].

Similar to our study, Wittchen et al. [39] found an increased association with CMD with higher age in a group of youth; for any anxiety disorder OR 1.30 (95 % CI 1.01–1.67; *p* < 0.05) while in Zimbabwe Langhaug et al. [36] did not find a significant association between increase in age and affective disorders in a sample of youth. Most literature from India have also shown older age groups to have a higher prevalence of psychiatric disorders; however, these studies are not specific to youth [20, 41]. However, evidence to show older age to be associated with CMD in females, but not in males, is limited. A longitudinal study over the course of 12 years, in the United States looking at youth from ages 9 to 21, reported an increased prevalence of depressive symptoms after puberty in females compared to males [51] but association of age with depressive symptoms was not explored. A review paper written in Australia by Rowe and Tonge [52] suggests that the age of onset of a CMD is probably linked with genetics, the nervous system and social development. It is possible that these change with age and differ between males and females, and may contribute to older age being a risk factor in females in our sample, as there is some literature to support this [9]. Further exploration with life events taking place in the transition-age, female youth group may be required.

Some studies have reported that discussing problems—usually studied as informal help-seeking behaviour or as a component of interpersonal relationship or social support [53]—may reduce the likelihood of physical and mental health problems [54, 55]. In our investigation, there was a decreased likelihood of CMD among those who are able to discuss problems, which was only evident in the female group (Table 2). This result is supported by evidence that females are more likely to benefit from social support compared to males [56–58]. A longitudinal study on 1,057 pairs of opposite-sex twins, which assessed social support and the effect on major depression, found, women had higher levels of social support compared to their twin counter-parts, and the existence of social support had only significantly protective effects in females but not in the males [56]. In a study of 141 Swiss adolescents, Frey et al. observed that though girls did not have a relatively bigger social support networks than boys, they received informal support from friends and family almost daily compared to boys. He suggested that females may be more in need of, and hence benefit from, communicating and relating to others compared to males [59].

Research on CMD among youth in India is an important priority given that youth are projected to form the largest proportion of the population group by 2030 [19]. The main clinical implications of the study are related to the influence of social support and abuse on youth mental health and that addressing these determinants would potentially reduce the population burden of CMD in youth. There is currently no literature on male abuse victims in India, the clinical implications of which may be profound if further studies investigate abuse among young males and the long-term consequences. Given the diversity of cultures and differences within India, it is important to conduct similar studies in different states to draw a representative picture of risk factors for CMD particularly among youth in India.
